# Exploration of the anti-insomnia mechanism of *Ganoderma* by central-peripheral multi-level interaction network analysis

**DOI:** 10.1186/s12866-021-02361-5

**Published:** 2021-10-29

**Authors:** Yu Qiu, Zhu-Jun Mao, Ye-Ping Ruan, Xin Zhang

**Affiliations:** School of Pharmaceutical Sciences, Zhejiang Chinese Medical University, Binwen Road 548, Binjiang District, Hangzhou, 310053 Zhejiang Province China

**Keywords:** *Ganoderma*, Insomnia, Systems pharmacology, Central, Peripheral

## Abstract

**Background:**

*Ganoderma* (*Lingzhi* in Chinese) has shown good clinical outcomes in the treatment of insomnia, restlessness, and palpitation. However, the mechanism by which *Ganoderma* ameliorates insomnia is unclear. We explored the mechanism of the anti-insomnia effect of *Ganoderma* using systems pharmacology from the perspective of central-peripheral multi-level interaction network analysis.

**Methods:**

The active components and central active components of *Ganoderma* were obtained from the TCMIP and TCMSP databases, then screened to determine their pharmacokinetic properties. The potential target genes of these components were identified using the Swiss Target Prediction and TCMSP databases. The results were matched with the insomnia target genes obtained from the GeneCards, OMIM, DisGeNET, and TCMIP databases. Overlapping targets were subjected to multi-level interaction network analysis and enrichment analysis using the STRING, Metascape, and BioGPS databases. The networks analysed were protein-protein interaction (PPI), drug-component-target gene, component-target gene-organ, and target gene-extended disease; we also performed gene ontology (GO) and Kyoto Encyclopedia of Genes and Genomes (KEGG) analyses.

**Results:**

In total, 34 sedative-hypnotic components (including 5 central active components) were identified, corresponding to 51 target genes. Multi-level interaction network analysis and enrichment analysis demonstrated that *Ganoderma* exerted an anti-insomnia effect via multiple central-peripheral mechanisms simultaneously, mainly by regulating cell apoptosis/survival and cytokine expression through core target genes such as TNF, CASP3, JUN, and HSP90αA1; it also affected immune regulation and apoptosis. Therefore, *Ganoderma* has potential as an adjuvant therapy for insomnia-related complications.

**Conclusion:**

*Ganoderma* exerts an anti-insomnia effect via complex central-peripheral multi-level interaction networks.

## Background

Insomnia is a common sleep disorder that is generally defined as dissatisfaction with sleep quantity or quality. The treatment of insomnia usually consumes substantial medical resources. Insomnia is experienced by 33–50% of the adult population. Its prevalence ranges from 10 to 15% in the general population [1]. An epidemiological study in China [2] showed that 45.4% of the respondents experienced varying degrees of insomnia in the previous month. Daytime dysfunction caused by insomnia includes fatigue, depression or irritation, physical discomfort, and cognitive impairment. It is also a common complication and trigger of cardiovascular, cerebrovascular, and mental diseases [3].

Compared with Western medicine, which relies on sedative and hypnotic drugs, traditional Chinese medicine (TCM) for insomnia has lower tolerance and dependence, as well as fewer adverse reactions; thus, it has become an important alternative therapy in East Asia, North America, Europe, and other regions [4]. *Ganoderma* is a medicinal mushroom that contains various pharmacological compounds. Medicinal *Ganoderma* is usually the dried fruiting body of *Ganoderma lucidum* (Leyss. ex Fr.) Karst or *Ganoderma sinense* Zhao, Xu et Zhang. According to the Chinese pharmacopoeia records, *Ganoderma* invigorates *Qi* (‘life energy’ or ‘life force’ in TCM); tranquilises the mind; and is used for the treatment of insomnia, palpitations, cough, and asthma [5]. To our knowledge, there have been few studies regarding the neuropharmacological activities of *Ganoderma*, including its sedative, hypnotic, neuroprotective, antinociceptive, analgesic, antiepileptic, and antidepressant effects [6]. Randomised controlled trials for insomnia have shown that *Ganoderma* can improve sleep quality and reduce the incidences of adverse effects and dependence [7, 8]. In animal studies, *Ganoderma* extract reduced sleep latency and prolonged sleep duration, which might be related to tumour necrosis factor (TNF) and *γ*-aminobutyric acid receptor activities [9, 10]. *Ganoderma* has anti-inflammatory, anti-oxidant, anti-hyperglycaemic, anti-ulcer, and immunostimulatory effects [11]. Therefore, *Ganoderma* can be used for the prevention and treatment of insomnia; the underlying mechanism warrants investigation.

Because *Ganoderma* contains large numbers of active components, which may interact with each other, it may have preventive and/or therapeutic effects against various diseases in multiple systems. This complex pharmacological network hampers systematic research regarding *Ganoderma*. Systematic pharmacology provides a new choice and direction for the study of drugs with complex pharmacological networks by integrating systems biology with pharmacology.

**Fig. 1:**
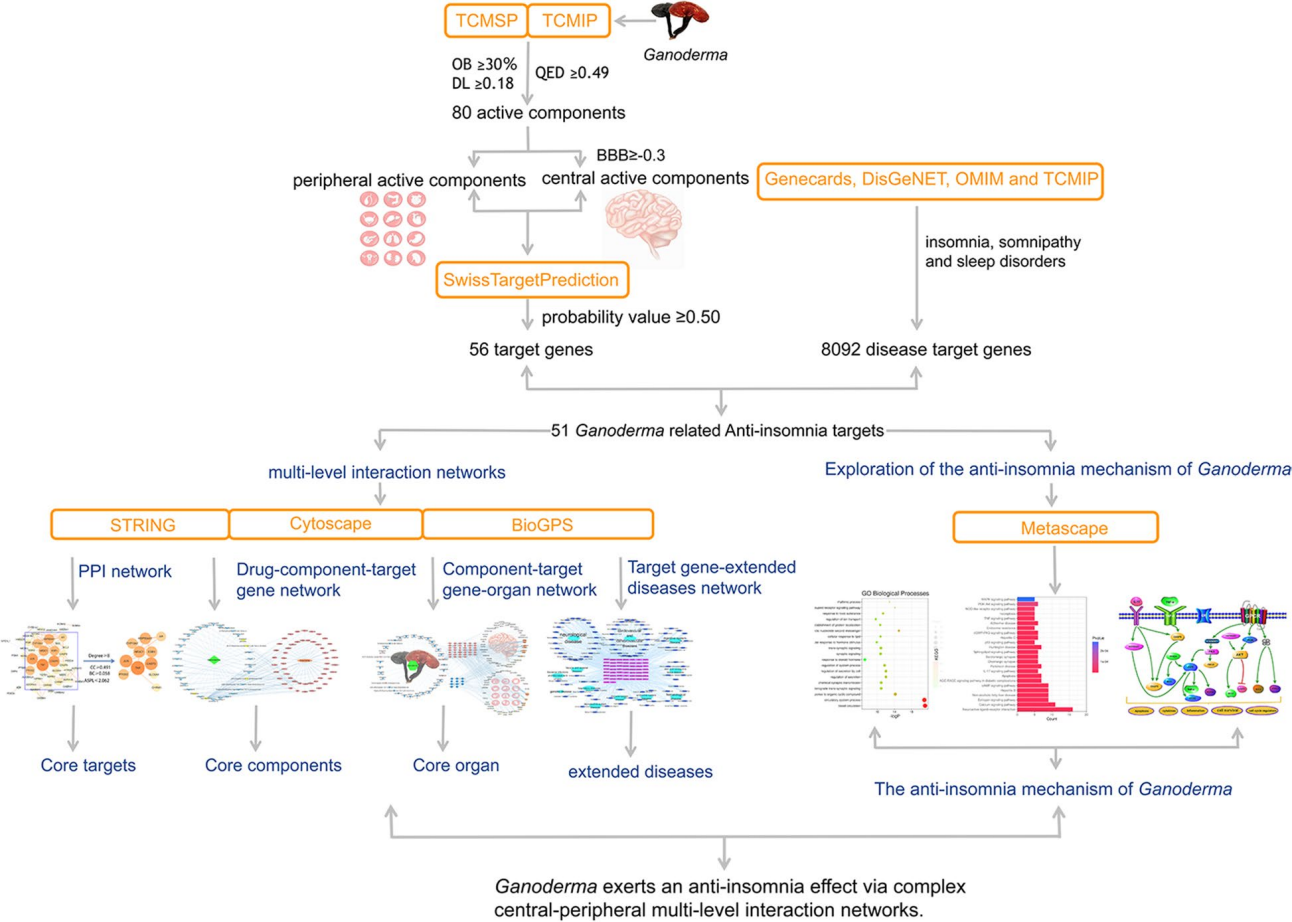
Diagram of experimental process. TCMSP: Traditional Chinese Medicine Systems Pharmacology Database and Analysis Platform; TCMIP: Integrative Pharmacology-based Research Platform of Traditional Chinese Medicine; OB: Oral bioavailability; DL: Drug-likeness; QED: Quantitative Estimation of Drug-likeness; BBB:blood-brain barrier

Here, we explored the sedative and hypnotic effects of *Ganoderma* to analyse the mechanism of its effects on insomnia. Because insomnia is complex and regulated by central and peripheral mechanisms, we first identified the anti-insomnia components of *Ganoderma*, isolated active components and central active components, classified the target genes by two-dimensional (2D) and three-dimensional (3D) similarity measurements, and matched the results with the insomnia target genes in multiple databases to identify overlapping targets. By using this process, multi-component, −target, −pathway, −organ, and -interaction networks were constructed. Compared with a single-level network analysis, which explains the mechanism from a single perspective, multi-level networks are more similar to in vivo pharmacodynamics. This is particularly relevant for insomnia, which is regulated by central and peripheral mechanisms. The experimental process is shown in Fig. 1.

## Methods

### Establishment of *Ganoderma* active-components dataset

The Integrative Pharmacology-based Research Platform of Traditional Chinese Medicine (TCMIP, http://www.tcmip.cn/) v. 2.0 is a data-mining platform that uses the database resources of the Encyclopedia of Traditional Chinese Medicine; it provides insights into the material basis and molecular mechanism of TCM efficacy. By inputting the Latin name of *Ganoderma* into the database, we retrieved all chemical constituents, then screened them by quantitative estimation of drug-likeness (QED score; calculated according to the Pipeline Pilot ADMET collection model, including aqueous solubility, blood brain barrier penetration, CYP450 2D6 inhibition, hepatotoxicity, human intestinal absorption, and plasma protein binding). The reported mean QED values for attractive and unattractive components in drug development were 0.67 and 0.49. The components of *Ganoderma* with moderate and good QED scores (QED ≥ 0.49) were retained [12].

The Traditional Chinese Medicine Systems Pharmacology Database and Analysis Platform (TCMSP, https://tcmspw.com/tcmsp.php) is an efficient database for systems pharmacology research regarding TCM. TCMSP was used to supplement the information regarding *Ganoderma* chemical composition. Oral bioavailability (OB) refers to the proportion of an orally administered drug that reaches the systemic circulation; this is a key indicator of the properties of bioactive molecules and drugs, and it has a high effect ratio. In this study, the oral bioavailability predicting model was supported by a dataset of 805 structurally diverse drugs with determination coefficients (*R*^2^) of 0.80 and standard errors of estimate of 0.31 for test sets; the model integrated P450, 3A4, and P-glycoprotein information. Drug-likeness (DL) represents the ‘drug-like’ degree of the target compound; this metric was used to remove chemically unsuitable compounds. TCMSP used the Tanimoto coefficient to calculate the drug-likeness index by comparing the target compound with all 6511 molecules in the DrugBank database (Eq. 1).

1 $$F\left(X,Y\right)=\frac{XY}{\left(\ {\left|X\right|}^2+{\left|Y\right|}^2- XY\right)\ }$$

where X represents the molecular properties of the compound in *Ganoderma*, and Y represents the mean molecular properties of all compounds in the DrugBank database (https://www.drugbank.ca).

To identify components that may be absorbed orally and exert curative effects, based on the mean value for all compounds in the DrugBank database, we selected the following threshold conditions for screening active components: OB ≥ 30% and DL ≥ 0.18.

The active components of *Ganoderma* identified by TCMIP and TCMSP were combined, the names were standardised in the PubChem database (https://pubchem.ncbi.nlm.nih.gov/) [13], and duplicates and invalid data were removed. Thus, the *Ganoderma* active component dataset was created. Because insomnia is closely related to the central nervous system, the central active components with a blood-brain barrier (BBB) score ≥ − 0.3 were extracted to facilitate exploration of the drug-disease relationship; higher scores indicate greater blood-brain barrier permeability. These were presumed to directly affect the central nervous system and used to explore the central-peripheral regulatory mechanism of *Ganoderma*.

### Prediction of target genes of *Ganoderma* active components

The SMILES string of the active components of *Ganoderma* or the SDF files of their molecular structures were searched in the PubChem database, then imported into the Swiss Target Prediction database (http:∥swisstargetprediction.ch/) [14]. The species was defined as *Homo sapiens*. Predictions were performed by searching for similar molecules, in 2D and 3D, among 376,342 compounds known to be experimentally active on an extended set of 3068 macromolecular targets. The 2D approach compares fingerprints describing each molecule; similarity is computed as the Tanimoto coefficient. In the 3D approach, molecules are represented by an 18-dimensional vector. The Manhattan distance is used to compare vectors (X and Y) describing two different molecules (Eq. 2). The final 3D similarity value between molecules I and j is computed, where *dij* is the smallest Manhattan distance among the 20 × 20 distances calculated over all possible conformations of each molecule (Eq. 3).

2 $$d={\textstyle\sum_{s=1}^{18}}\left|Xs-Ys\right|$$

3 $$1/\left(1+\frac{1}{18}{d}_{ij}\right)$$

The potential target genes of *Ganoderma* active components could be predicted after running [15]. The target genes with high credibility were screened with a probability value ≥0.50 as the threshold. For active components without results under the screening conditions, the MOL number was input into the TCMSP database to supplement the target gene information. In this study, TCMSP used the systems drug targeting model, based on a random forest and support vector machine method, to identify potential therapeutic targets of candidate compounds. The training set for the systems drug targeting model included 6511 drug molecules and almost 4000 proteins that interact with drug molecules in the DrugBank database. The results indicated good ability to predict drug-target interactions; the consistency, sensitivity, and specificity values were 82.83, 81.33, and 93.62%, respectively.

The active components and target genes were integrated to establish a dataset of the effective components and corresponding target genes of *Ganoderma*. The relationships between the components and target genes were input into Cytoscape (http://www.Cytoscape.org/) for visual analysis. The attributes of the components were classified as the source node, the attributes of target genes were classified as target nodes, the attributes of node type were classified as interaction type, and treated the network as undirected.

### Recognition of disease target genes based on multiple databases

To identify potential insomnia target genes, we used the keywords ‘insomnia’, ‘somnipathy’, and ‘sleep disorders’ to search the GeneCards (https://www.genecards.org/) [16], OMIM (https://omim.org/) [17], DisGeNET (https://www.disgenet.org/) [18], and TCMIP (https://www.tcmip.cn/) [12] databases. We merged the database findings and deleted repeated target genes, thus producing insomnia-related disease target genes.

### Construction of overlapping data of drugs and disease target genes

The active component target genes and insomnia target genes of *Ganoderma* were input into the Jvenn online tool (http://jvenn.toulouse.inra.fr/app/index.html). This tool was used to create a Venn diagram to assess the intersections of target genes between *Ganoderma* and insomnia, or the potential target genes of *Ganoderma* for insomnia treatment. This facilitated PPI network analysis and the construction of a multidimensional network.

### Construction of the PPI network

The STRING database (https://string-db.org/) [19] contains known protein interactions, enabling the construction of PPI networks. To assess the expression of intersection target genes, the potential target genes of *Ganoderma* for insomnia treatment were imported into the STRING database. The interaction credibility is determined by the confidence level (highest confidence, score ≥ 0.9; high confidence or better, score ≥ 0.7; medium confidence or better, score ≥ 0.4; and low confidence or better, score ≥ 0.15). In this study, the minimum required score was set to 0.4. The species was set as *Homo sapiens*. Free protein was removed. The PPI network of potential target genes for insomnia in *Ganoderma* was obtained and input into Cytoscape for visualisation. The complex network relationship was analysed using the AnalysisNetwork module; the following topological parameters were obtained: average shortest path length (ASPL), betweenness centrality (BC), closeness centrality (CC), and degree. The degree of each node represents the number of other nodes to which it is directly connected; BC refers to the number of times a node passes through the shortest path between any two other nodes; CC represents the reciprocal of the mean distance to all other nodes; and ASPL represents the mean of the shortest path between any two nodes. Larger degree, closeness centrality, and betweenness centrality values are associated with smaller average shortest path length values and stronger node centrality values. Using these parameters, the core proteins were identified by digitising the complex network relationship.

### Construction of the drug-component-target gene network

The effective component-target gene data of *Ganoderma* were input into Excel to determine the intersection target genes screened by PPI; components that exhibited no relationships with the intersection target gene were deleted. Thus, components of *Ganoderma* with potential sedative and hypnotic effects were obtained. The correlations among components, target genes, diseases, and drugs were input into Cytoscape to construct a visual drug-component-target gene network map. By analysing the degree of the target gene, it was determined that the target gene was jointly affected by several effective components, based on which the credibility of intervention by the target gene was examined. By analysing the connectivities of the active components, we determined which active components simultaneously acted on the target gene, then examined their biological activities.

### Pathway enrichment analysis of sedative and hypnotic target genes

The sedative-hypnotic target genes screened by PPI were integrated and input into the online platform Metascape (http://metascape.org/) [20] for enrichment analysis of GO biological processes and KEGG pathways [21]. *P*-values were calculated based on the cumulative hypergeometric distribution; they were corrected by the Benjamini-Hochberg method. The most representative enrichment analysis results were selected with *P* < 0.01 [22]. Eventually, those results were visualized by bioinformatics online tool (http://www.bioinformatics.com.cn).

### Construction of the component-target gene-organ network

The BioGPS database (http://biogps.org/#goto=welcome) [23] is a centralised gene portal for aggregating distributed gene annotation resources; targets can be located by querying the expression patterns of genes in cells or tissues. In this study, the BioGPS database was used to identify sedative and hypnotic targets of *Ganoderma*. After inputting the targets, human was chosen as the species and ‘GeneAtlas U133A, gcrma’ in the default dataset was selected. After correlation sorting and removal of meaningless results, the positioning results were integrated with the sedative and hypnotic components-target gene dataset; Cytoscape was used to draw the *Ganoderma* positioning network map. Because the brain regulates sleep, organs or tissues related to the brain were listed separately in the organ-mapping diagram to explore the relationships of different brain functional areas with the improvement of insomnia and to examine the target organs of the central-peripheral mechanism.

### Construction of the target gene-extended disease network

To explore the pharmacological effects of *Ganoderma* on insomnia, the sedative and hypnotic target genes of *Ganoderma* were reversely enriched in the DisGeNET database based on the Metascape online platform (*P* < 0.01). The data were input into Cytoscape for visualisation to explore the therapeutic effect of *Ganoderma* on insomnia, then determine its utility as a treatment or adjuvant therapy for insomnia complications.

## Results

### *Ganoderma* active-component dataset

Using the TCMSP and TCMIP databases, duplicate items were deleted, and the names were standardised. Based on their pharmacokinetics (OB, DL, and BBB), 80 active components of *Ganoderma* were obtained, including 16 potential central active components (BBB ≥ − 0.3) (Table 1). *Ganoderma* contained a large number of active components, mostly triterpenoids (ganoderic acid, ganoderma alcohols, and ganoderma aldehydes) and sterols.

**Table 1:** Information of potential central active components

PubChem CID	chemical name	Molecular Formula	BBB	OB
444,679	Ergosterol	C28H44O	1.66	14.29
56,676,695	(24xi)-Ergosta-4,6,8(14),22-tetraene-3-one	C28H40O	1.15	48.32
222,284	Beta-Sitosterol	C29H50O	0.99	36.91
5,283,669	Stellasterol	C28H46O	0.98	43.51
101,449,382	Ergosta-7,22-diene-3beta-yl pentadecanoa	C43H74O2	0.72	38.25
69,888,957	Ergosta-7,22-diene-3beta-ol palmitate	C44H76O2	0.63	37.60
6,449,869	Ergosta-7,22-dien-3-yl linoleate	C46H76O2	0.53	45.11
11,177,299	Ganodesterone	C28H40O2	0.47	47.86
5,351,516	Ergosterol peroxide	C28H44O3	0.43	44.39
21,159,042	26,27-Dihydroxy-Lanosta-7,9(11),24-Trien-3,16-Dione	C30H44O4	0.13	28.95
11,048,424	Lucialdehyde A	C30H46O2	0.13	44.78
3,001,811	Ganodermanontriol	C30H48O4	0.07	28.92
471,007	Ganoderiol B	C30H46O4	−0.03	28.95
73,082,616	Ganoderol A	C30H46O2	−0.04	44.69
101,602,260	(22S)-22beta-Acetoxy-3alpha,15alpha-dihydroxy-5alpha-lanosta-7,9(11),24-trien-26-oic acid	C32H48O6	−0.06	37.64
68,018,655	Ganoderic acid Z	C30H48O3	−0.19	37.67

### Target genes of *Ganoderma* active components

**Fig. 2:**
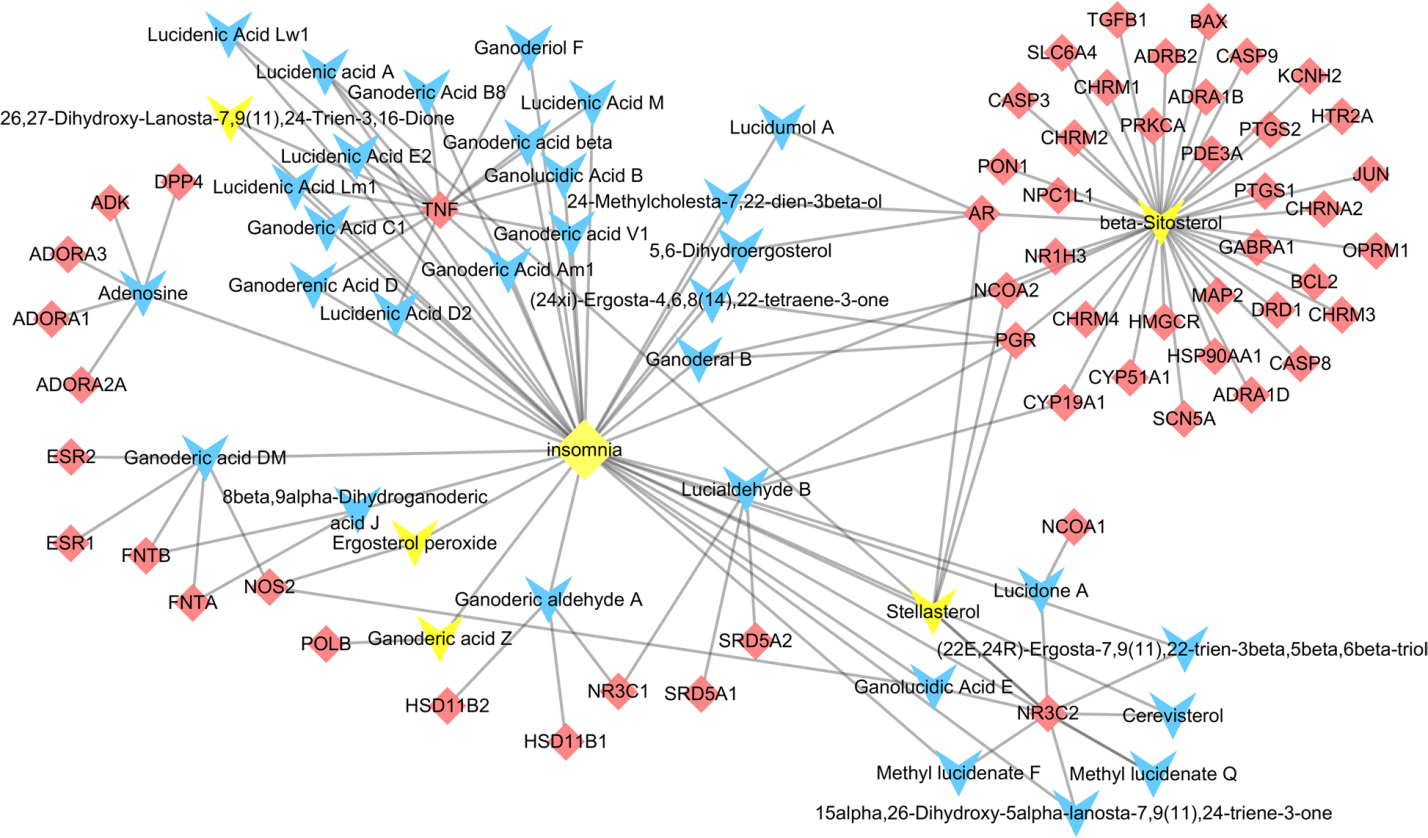
Target gene network of active components. The blue arrows represent the active components; the yellow arrows represent the central active components; the yellow diamonds represent diseases; the red diamonds represent target genes

Data from the Swiss Target Prediction tool and the TCMSP database indicated that 36 of the 80 active components had 56 target genes with high reliability (Fig. 2). The target genes and active components intersected, and the same active component could correspond to multiple target genes. For example, beta-sitosterol corresponded to 37 target genes. Beta-sitosterol has cholesterol-reduction, anti-inflammatory, and antitumour activities [24]; it may have an important role in the effects of *Ganoderma*. Beta-sitosterol extracted from herbs has sedative-hypnotic potential [25, 26]. In this study, beta-sitosterol showed good blood-brain barrier permeability (0.99), indicating that it comprises a potential central active component. We speculate that this *Ganoderma* component regulates the insomnia network.

The same target gene could be affected simultaneously by multiple active components. *Ganoderma* triterpenes had several physiological activities, including antitumour, liver protection, anti-human immunodeficiency virus, and cholesterol reduction [27]. TNF was jointly affected by 15 components, mainly triterpenoids, of *Ganoderma*; thus, *Ganoderma* may participate in sleep regulation by affecting TNF. Sleep is closely related to immunity. Sleep deprivation affects metabolism and increases the secretion of C-reactive protein, TNF, and interleukin-6 [28]; TNF activity increases non-rapid eye movement sleep [29]. These results will facilitate the improvement of *Ganoderma* pharmacological activity. In summary, the active components of *Ganoderma* are complex and interact with each other.

### Disease target genes in multi-source databases

In total, 8092 target genes were obtained by integrating disease target genes from the GeneCards, DisGeNET, OMIM, and TCMIP databases. The mechanism of insomnia was complex, involving large numbers of target genes.

### Intersections of drugs and disease target genes

**Fig. 3:**
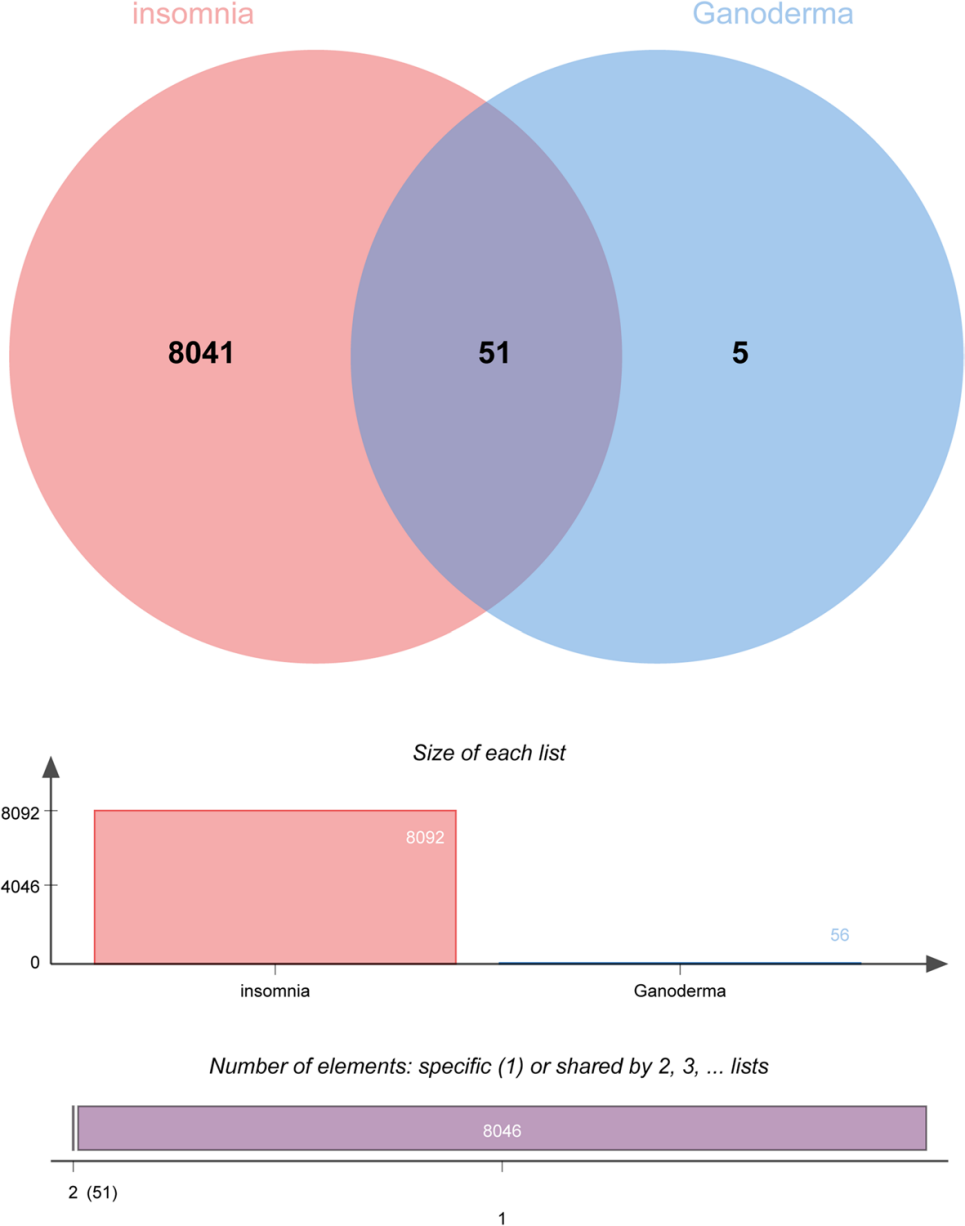
Venn diagram of drug-disease target genes. The red region represents the insomnia target genes, and the blue region represents the Ganoderma target genes

A Venn diagram was created by crossing the target genes of active components of *Ganoderma* with insomnia-related targets (Fig. 3). There were 51 targets for the treatment of insomnia, constituting 91.07% of the total. *Ganoderma* has therapeutic potential for insomnia; clarification of its anti-insomnia mechanism is important for clinical application and drug development.

### PPI network

The potential target genes of *Ganoderma* for insomnia were imported into the STRING database for PPI network analysis, using nodes to represent the target genes and edges to represent the relationships between target genes. The number of nodes in the PPI network of *Ganoderma* target genes was 51 (without free targets); the number of edges was 194. The mean node degree was 7.61 and the mean local clustering coefficient was 0.554. Network data analysis showed close relationships among *Ganoderma* target genes in insomnia treatment.

**Fig. 4:**
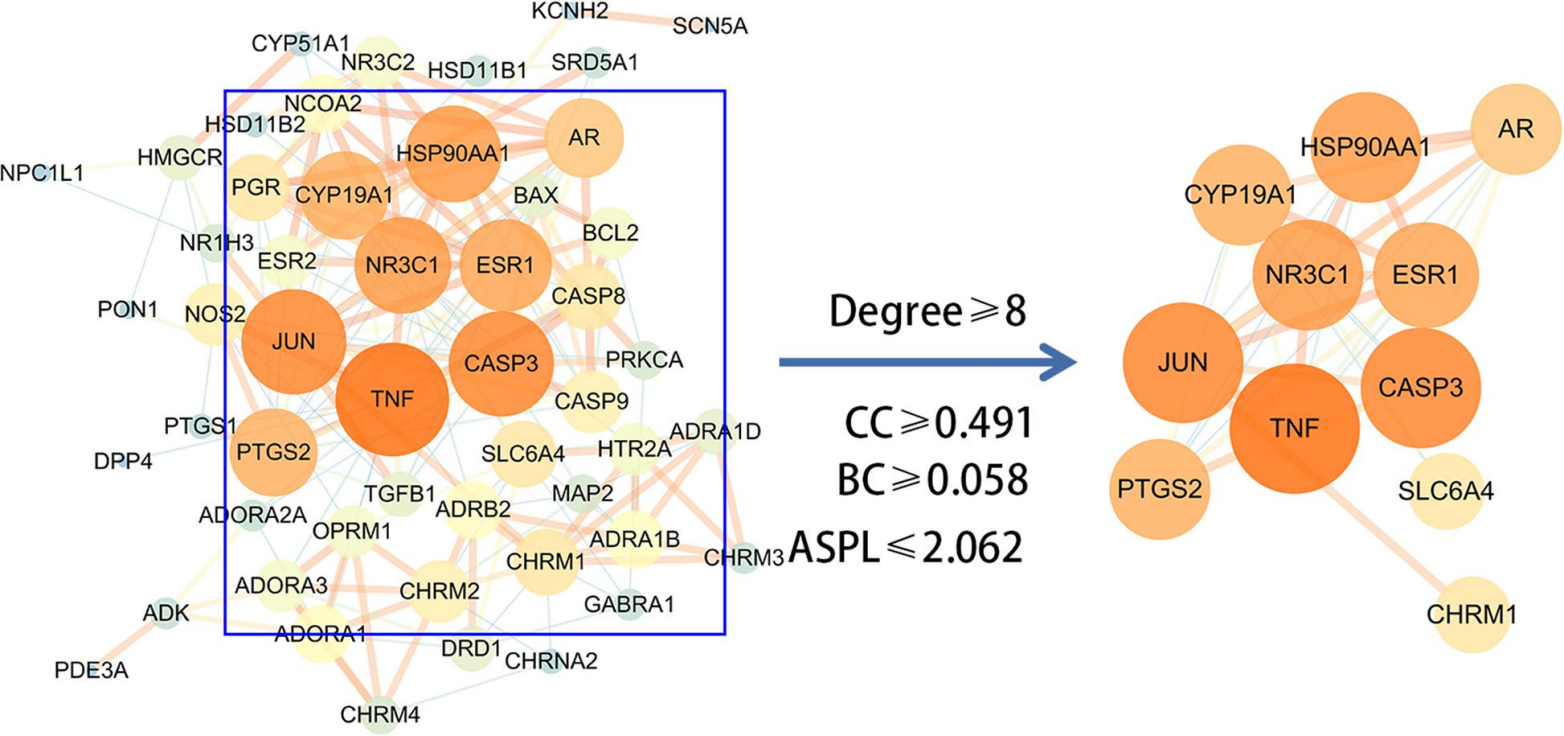
PPI network and core protein network. All node color and size depended on the Degree value. Topological Screening of Proteins with Degree≥8(The average value of Degree), 11 core proteins with median topological feature values above/below the median were identified. CC: Closeness Centrality; BC: Betweenness Centrality; ASPL: Average Shortest Path Length

To identify core target genes in the PPI network, the network data were input into Cytoscape for visualisation (Fig. 4) and topology analysis (Table 2). The central properties of target genes were evaluated by topological analysis. TNF, caspase-3 (CASP3), transcription factor AP-1 (JUN), glucocorticoid receptor (NR3C1), heat shock protein 90-alpha (HSP90αA1), estrogen receptor 1, prostaglandin G/H synthase 2, cytochrome P450 family 19 subfamily A member 1, androgen receptor, and others were core proteins in the PPI network; they had important roles in the regulatory network.

**Table 2:** Topological analysis of PPI network (top 20 sorted by degree)

Targets	Degree	ASPL	BC	CC
TNF	21	1.720	0.236	0.581
CASP3	19	1.780	0.090	0.562
JUN	19	1.720	0.120	0.581
NR3C1	17	1.780	0.132	0.562
HSP90αA1	17	1.880	0.120	0.532
ESR1	16	1.940	0.029	0.515
PTGS2	15	1.960	0.020	0.510
CYP19A1	15	1.880	0.094	0.532
AR	13	2.000	0.019	0.500
SLC6A4	10	2.120	0.066	0.472
CHRM1	10	2.160	0.079	0.463
PGR	10	2.180	0.003	0.459
CASP8	10	2.160	0.003	0.463
CHRM2	9	2.440	0.031	0.410
CASP9	9	2.180	0.003	0.459
NOS2	9	2.100	0.030	0.476
ADORA1	8	2.260	0.041	0.442
ADRA1B	8	2.540	0.016	0.394
ADRB2	8	2.080	0.033	0.481
NCOA2	8	2.360	0.005	0.424

### Drug-component-target gene network

**Fig. 5:**
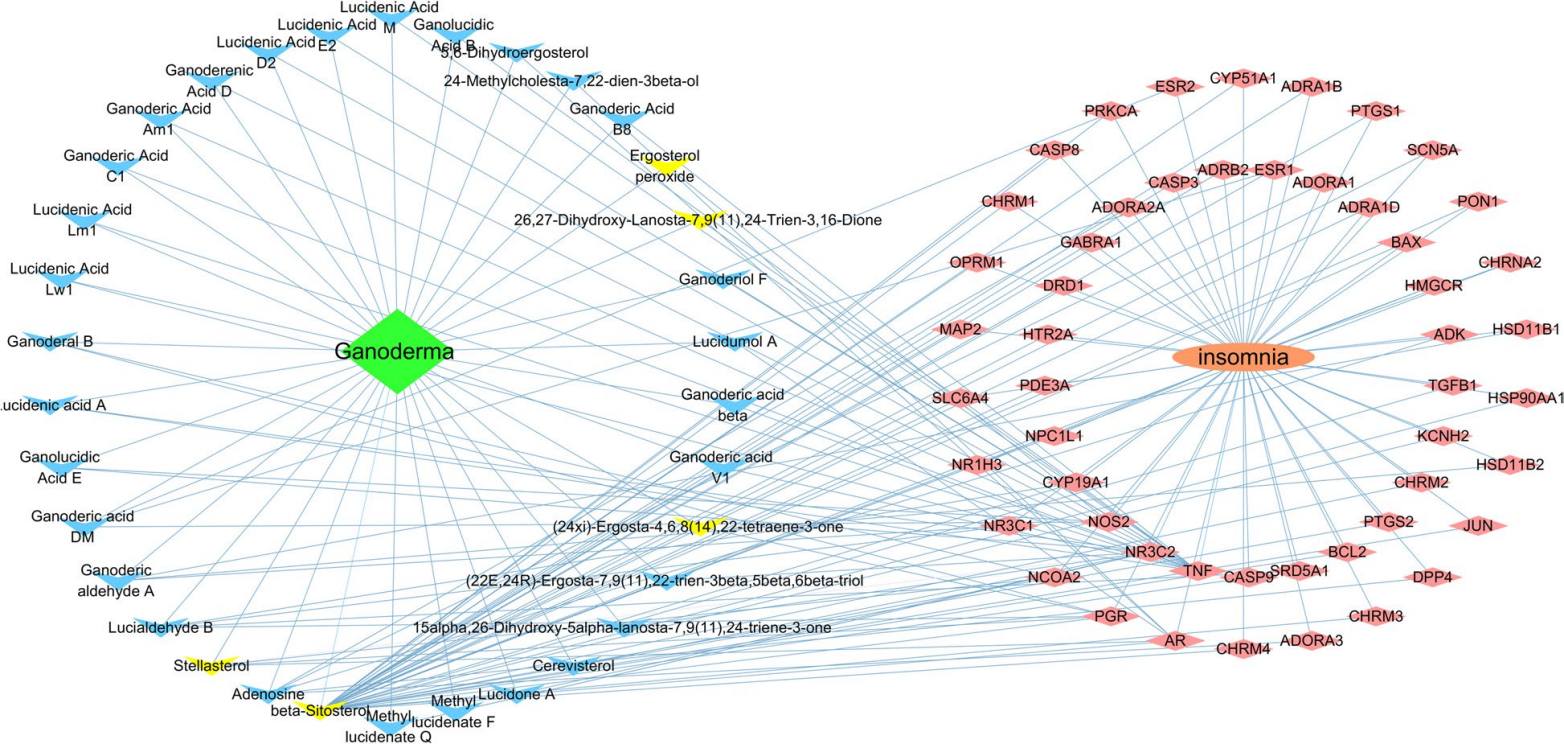
Drug-component-target gene network. The green rhombus represents Ganoderma; the blue arrow represents the active components; the yellow arrow represents the central active components; the red rhombus represents the target genes; and the red ellipse represents insomnia

Using Excel and visualisation in Cytoscape, the drug-component-target gene network of *Ganoderma* for insomnia treatment was obtained (Fig. 5). The network included 51 sedative-hypnotic target genes and 34 sedative-hypnotic components (including 5 central sedative-hypnotic components) of *Ganoderma*.

TNF was the target gene with the highest degree value of 16, followed by 10 for mineralocorticoid receptor (NR3C2), 6 for androgen receptor and progesterone receptor, and 4 for nitric oxide synthase 2 and nuclear receptor coactivator 2. These results indicate that the target gene is affected by multiple components, and the reliability of the intervention is high.

Among the sedative and hypnotic components, the component with the highest degree value was beta-sitosterol (38), followed by adenosine (6), stellasterol (5), lucialdehyde B (5), ganoderic aldehyde A (4), and ganoderic acid DM (4). These components had high connectivity, suggesting that they interfere with the development of insomnia through multiple target genes and pathways.

### GO enrichment analysis

**Fig. 6:**
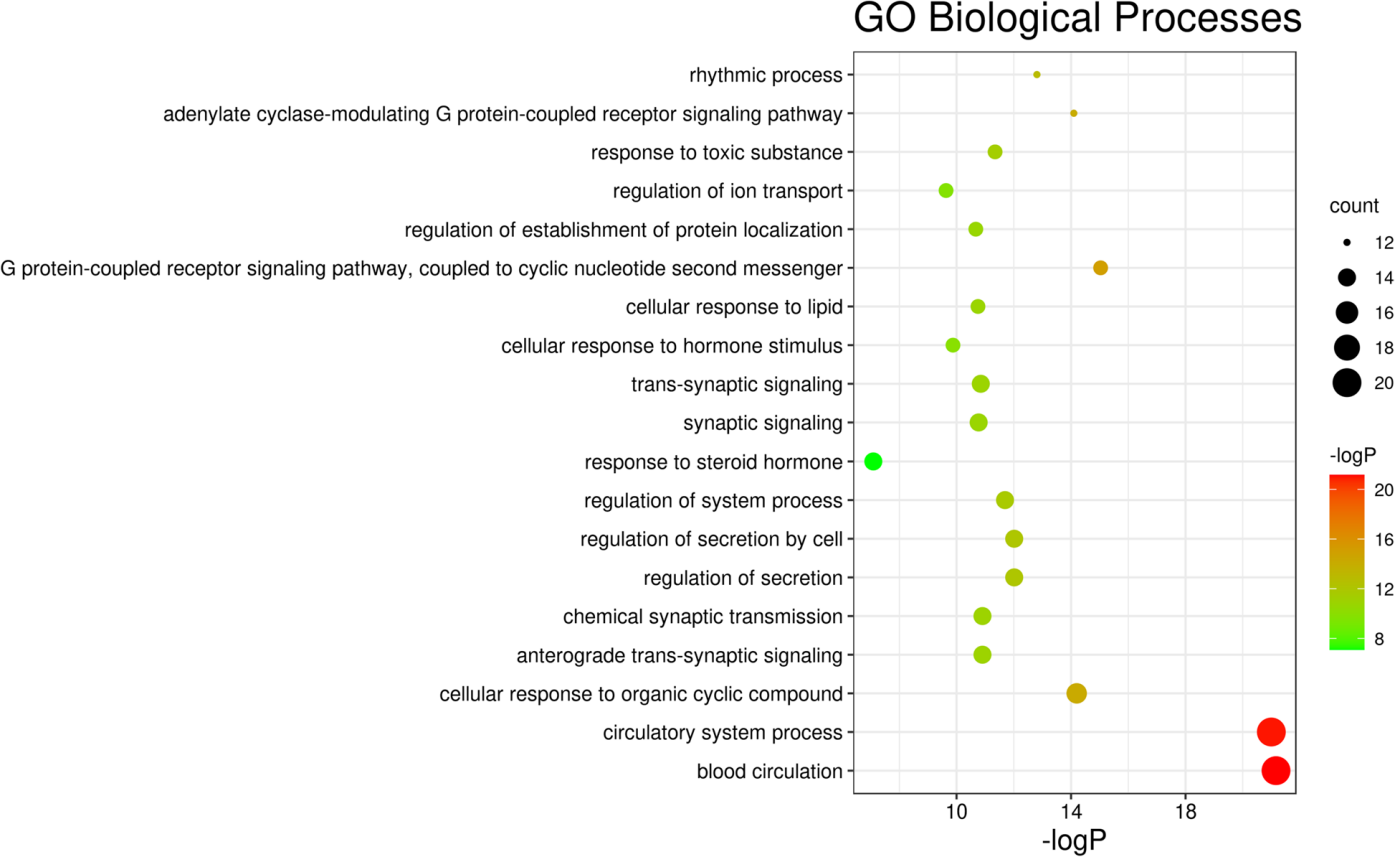
Bubble diagram of GO Biological Processes

Using the Metascape online platform for GO enrichment analysis, 694 representative functional clusters were obtained. According to the number of target genes involved, the top 20 enrichment results were retained for analysis (Fig. 6). The GO enrichment results were concentrated in cyclic metabolism, synaptic signalling, cell secretion and response, and G protein-coupled receptor signalling pathway.

Cyclic metabolism included blood circulation (GO: 0008015), the circulatory system process (GO: 0003013), and regulation of the system process (GO: 0044057). Synaptic signals included chemical synaptic transmission (GO: 0007268), anterograde trans-synaptic signalling (GO: 0098916), trans-synaptic signalling (GO: 0099537), and synaptic signalling (GO: 0099536). Cell secretion and response included regulation of secretion by cells (GO: 1903530), regulation of secretion (GO:0051046), response to steroid hormone (GO: 0048545), cellular response to lipid (GO: 0071396), and cellular response to hormone stimulation (GO: 0032870). The G protein-coupled receptor signalling pathway included G protein-coupled receptor signalling pathway, coupled to cyclic nucleotide second messenger (GO: 0007187), and adenylate cyclase-modulating G protein-coupled receptor signalling pathway (GO: 0007188). The rhythmic process (GO: 0048511) is closely related to sleep regulation, suggesting that *Ganoderma* improves insomnia by affecting biological rhythms.

### KEGG enrichment analysis

**Fig. 7:**
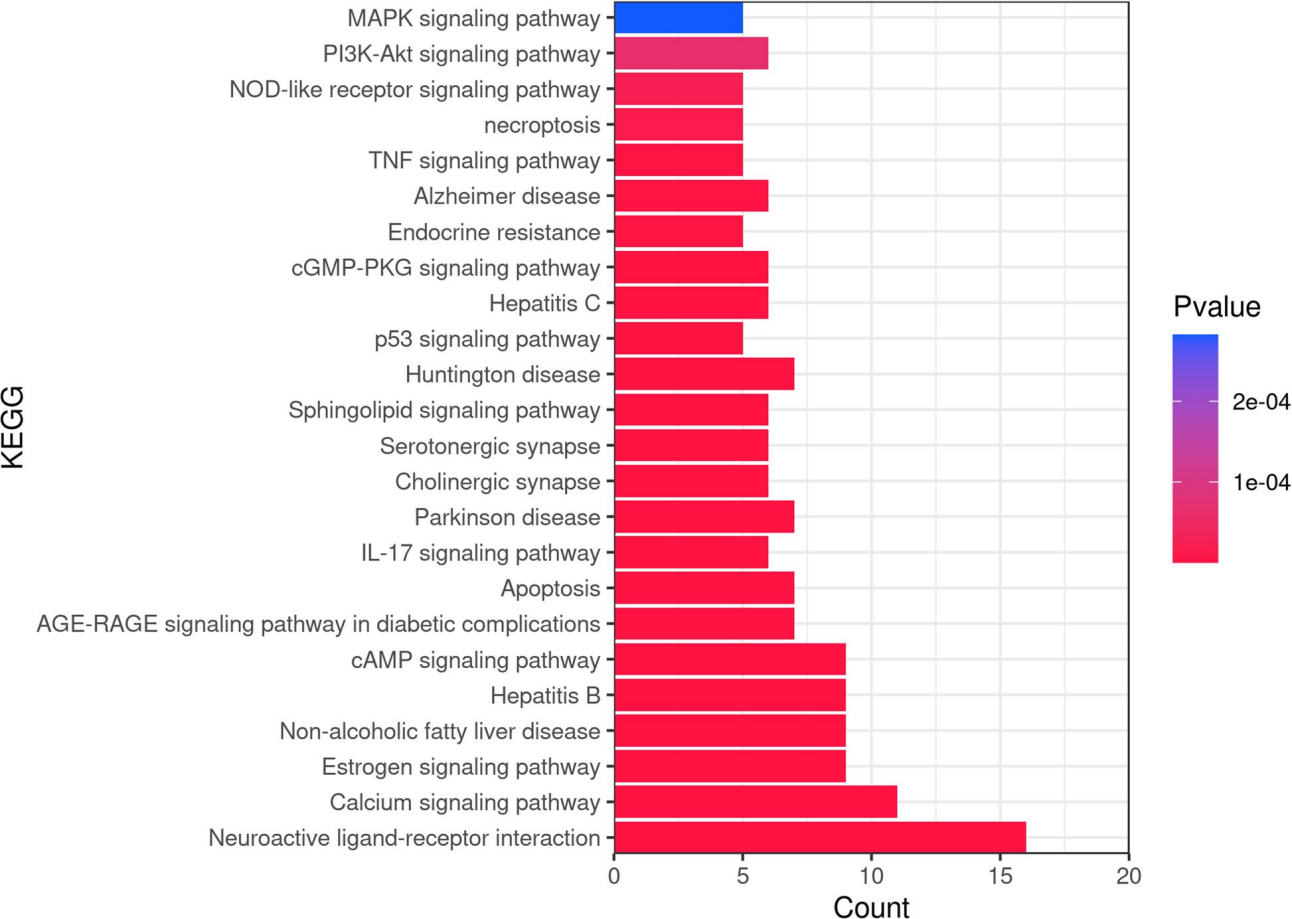
KEGG enrichment pathway

**Fig. 8:**
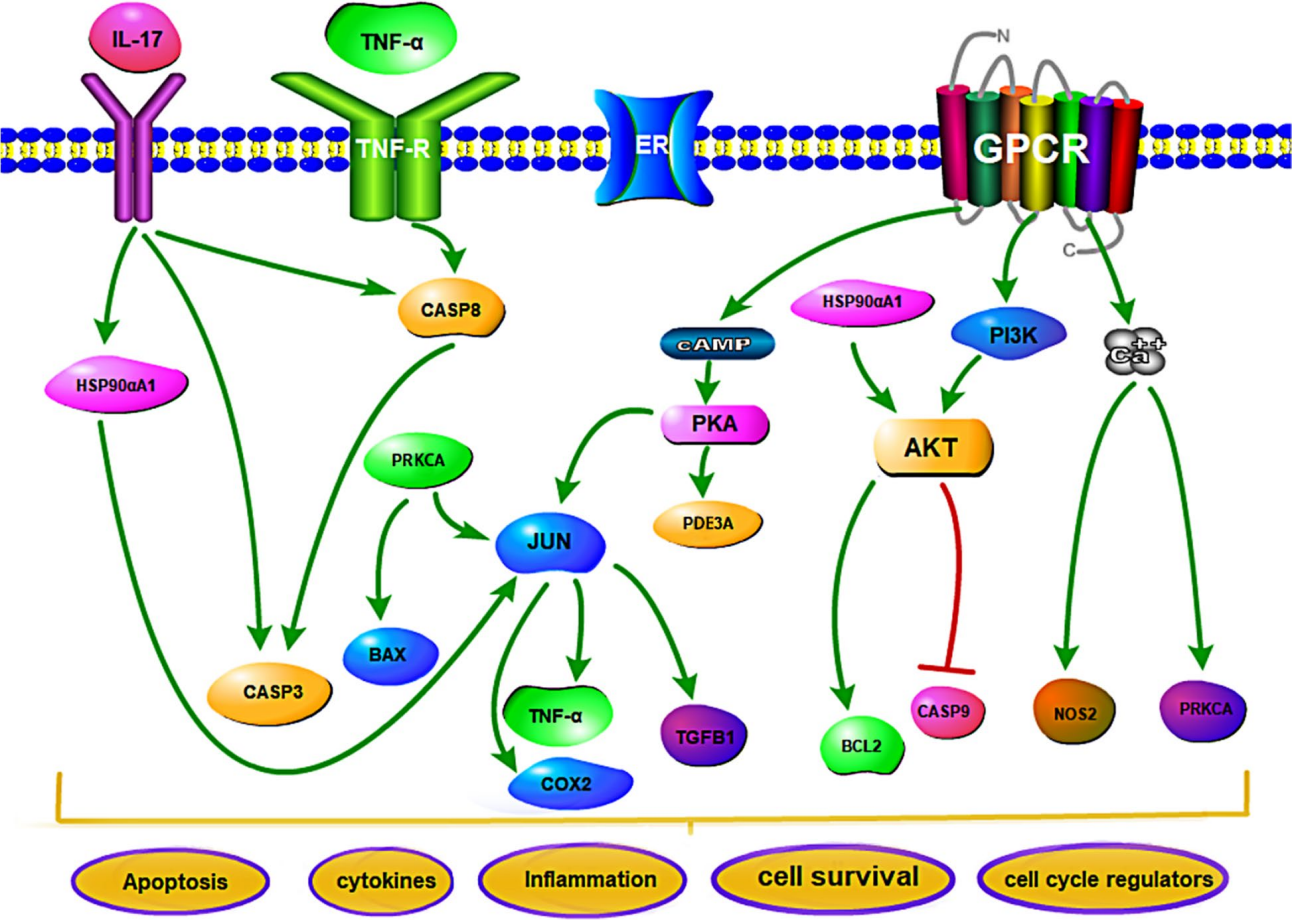
Mechanism diagram of *Ganoderma* treating insomnia. IL-17: Interleukin 17; TNF-α:Tumor necrosis factor-alpha; TNF-R: Tumor necrosis factor-Receptor; ER: Estrogen receptor; GPCR: G protein-coupled receptor; HSP90αA1: Heat shock protein 90-alpha; CASP: Caspase; PRKCA: Protein kinase C alpha type; BAX: Apoptosis regulator BAX; JUN: Transcription factor AP-1; COX2: Cytochrome c oxidase subunit 2; TGFB1: Transforming growth factor beta-1; cAMP: Cyclic adenosine monophosphate; PKA: Protein kinase A; PDE3A: Phosphodiesterase 3A; PI3K: Phosphatidylinositol 3-kinase; AKT(PKB): Kinase-protein kinase B; BCL2: B-cell lymphoma 2; NOS2: Nitric oxide synthase

Using the Metascape online platform for KEGG enrichment analysis, 93 representative functional clusters were identified after the removal of signalling pathways that exhibited weak correlations with insomnia; these included pathways in cancer (hsa05200), tuberculosis (hsa05152), toxoplasmosis (hsa05145), and small-cell lung cancer (hsa05222). According to the number of target genes involved in sorting, and after the retention of results with ≥5 target genes, 24 targets were obtained (Fig. 7). Next, seven pathways with high analytical values were selected for sorting; the pathway diagram of *Ganoderma* for insomnia treatment is shown in Fig. 8. The largest number of targets was involved in the neuroactive ligand-receptor interaction signalling pathway (hsa04080), which is a collection of plasma membrane receptors and ligands related to intracellular and extracellular signalling pathways, suggesting that *Ganoderma* affects receptor–ligand interactions. Through analyses of the calcium signalling pathway (hsa04020), cyclic adenosine monophosphate (cAMP) signalling pathway (hsa04024), apoptosis (hsa04210), interleukin-17 signalling pathway (hsa04657), phosphatidylinositol 3-kinase-protein kinase B (PI3K-Akt) signalling pathway (hsa04151), TNF signalling pathway (hsa04668), and other pathways, we found that TNF, JUN, HSP90αA1, and other core target genes ameliorate insomnia by interfering with the above target genes related to apoptosis/survival, cell cycle regulation, cytokines, and inflammation. In addition, cholinergic synapse (hsa04725) and serotonergic synapse (hsa04726) were notable pathways because they are reportedly related to insomnia and the regulation of sleep and arousal [30, 31].

Several neurodegenerative disease-related pathways were detected, such as Huntington disease (hsa05016), Parkinson disease (hsa05012), and Alzheimer disease (hsa05010); there is often a bidirectional relationship between the above neurodegenerative diseases and insomnia [32]. Hepatitis B, hepatitis C, non-alcoholic fatty liver disease, and other liver-related disease pathways were also enriched. A considerable proportion of hepatitis B patients have insomnia [33]. The relationship between liver and insomnia is weak, and the mechanism is unknown.

### Component-target gene-organ network

**Fig. 9:**
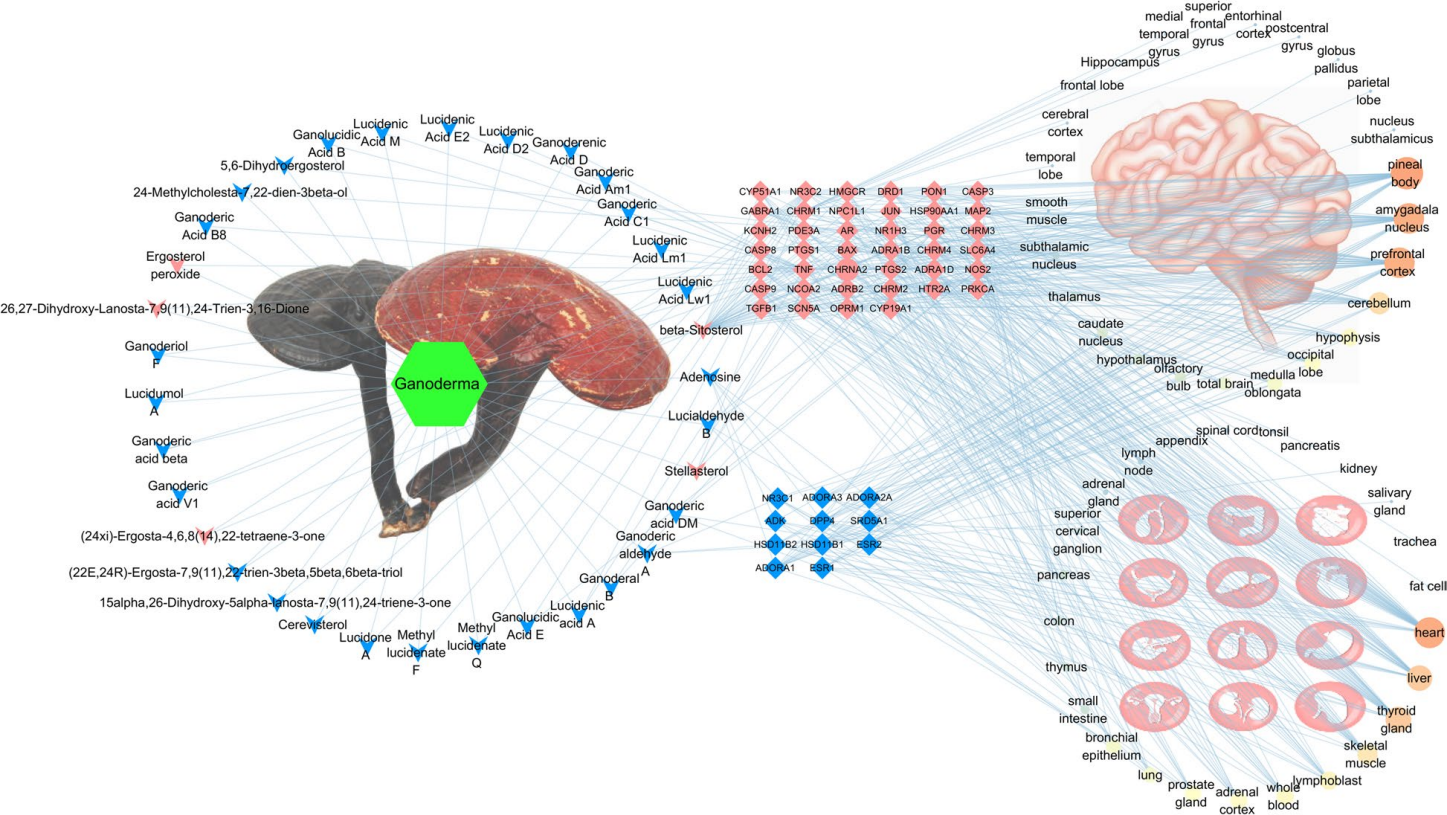
Component-target gene-organ network. The arrow represents the sedative and hypnotic components of *Ganoderma*: the red arrow represents the central active component; the red rhombus represents the target genes corresponding to the central active components; the blue rhombus represents other target genes; the right circle represents the organ. The node size and color depend on Degree

Organ localisation of *Ganoderma* sedative and hypnotic target genes was performed using the BioGPS database. The results were integrated with the component-target gene dataset, then input into Cytoscape for visual mapping and analysis (Fig. 9). Although the central active components of *Ganoderma* comprised only a small portion of the total, the number of target genes affected by *Ganoderma* was greater than the number of genes affected by other components. *Ganoderma* may affect the central nervous system in the treatment of insomnia; beta-sitosterol made the greatest contribution to this process. However, the target organs of the central active components included—but were not limited to—the central organs. Furthermore, other target genes were also highly expressed in the central and peripheral organs. Therefore, we speculate that *Ganoderma* exerts its anti-insomnia effects by influencing the expression of the same target genes in multiple organs simultaneously (i.e., a central-peripheral mechanism).

In the central mechanism, *Ganoderma* mainly affected the pineal body, amygdala nucleus, prefrontal cortex, cerebellum, and other regions. The pineal body is an important regulatory hub of the human biological clock, to which melatonin and 5-hydroxytryptamine are closely related [34]. The amygdala nucleus and prefrontal cortex are important functional areas of emotion control and have roles in sleep regulation [35]. In the peripheral mechanism, the heart, thyroid gland, and liver exhibited more target genes. Cardiovascular disease is closely related to circadian rhythm disorder [36]; TCM theory postulates a close relationship among the heart, brain, and spirit. Our results provide a scientific explanation for ‘heart and brain jointly dominate the spirit.’ Changes in thyroid gland function affect the peripheral biological clock [37], possibly improving insomnia. The liver metabolises vitamins and hormones [38]. To our knowledge, there have been few modern studies regarding the relationship between liver and insomnia, and the underlying mechanism warrants further investigation.

### Target gene-extended disease network

**Fig. 10:**
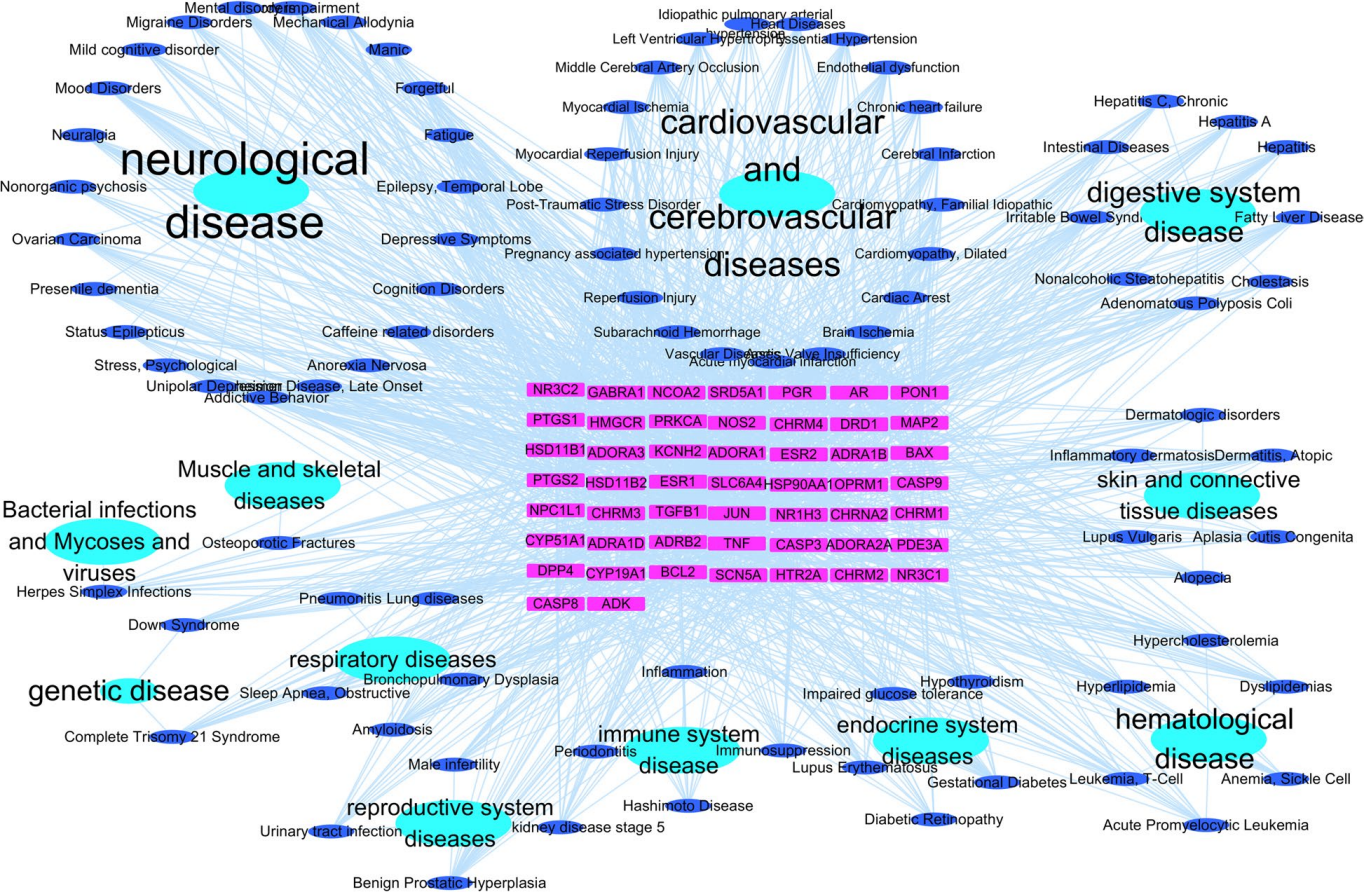
Target gene-extended diseases network. The purple rectangle represents the target gene; the blue circle represents the disease; the cyan circle represents the disease type

Reverse disease enrichment of *Ganoderma* sedative hypnotic target genes was carried out using the Metascape online platform; it yielded 1874 enrichment values. Results with > 10 enriched target genes were selected for analysis, and less valuable results were deleted (e.g., 48 tumour-related diseases). The appropriate results were retained and entered into Cytoscape for visualisation (Fig. 10). Neurological, cardiovascular and cerebrovascular, and digestive system diseases were the most common diseases.

## Discussion

Sleep is important for maintenance of normal physiological and psychological activities. Disturbance of the natural sleep rhythm can cause insomnia and various pathophysiological changes. Central pacemaker neurons are the main nodes of rhythm regulation, driving the biological clock of peripheral tissues. They jointly regulate the sleep rhythm process; thus, the development and formation of insomnia are affected by the central-peripheral mechanism [39]. Current drugs for insomnia include benzodiazepine receptor agonists, melatonin receptor agonists, orexin receptor antagonists, and antidepressants with sedative and hypnotic effects. They typically act on specific receptors (e.g., γ-aminobutyric acid receptor A and melatonin MT receptor), but these drugs can cause dependence and adverse reactions [40].

*Ganoderma*, a commonly used Chinese medicine for the treatment of insomnia, is rich in various active components. In this study, we identified 80 active components, mainly triterpenoids and sterols. We set a high screening threshold (probability value ≥0.50) in Swiss Target Prediction and TCMSP; we found 36 components corresponding to 56 target genes (34 sedative-hypnotic components corresponding to 51 target genes), including 5 central active components. The pharmacological action of *Ganoderma* was not limited to a single receptor or organ; it affected related proteins in multiple tissues or organs. Thus, *Ganoderma* exerts its pharmacological effects by simultaneously affecting multiple central-peripheral mechanisms, whereas commonly used insomnia drugs typically affect a single receptor.

Analysis of the drug-component-target gene network showed that the components beta-sitosterol, adenosine, stellasterol, lucialdehyde B, ganoderic aldehyde A, and ganoderic acid DM had high degree values, indicating that they had important roles in the regulatory network. Beta-sitosterol and stellasterol have a wide range of physiological functions. Thus far, research regarding their pharmacological activities mainly focuses on lowering cholesterol and blood lipid levels, as well as their anti-inflammatory effects. Beta-sitosterol and related fatty acids have anti-anxiety and sedative effects [25], but the underlying mechanisms are unclear. However, our results suggest that beta-sitosterol has great potential in sedation and hypnosis; moreover, *Ganoderma* has high sterol content [41]. Therefore, regardless of component activity or content, *Ganoderma* sterols may have important roles in sedation and hypnosis. Adenosine, an active component of *Ganoderma*, has a role in sleep-wake regulation. Adenosine is a key signal molecule in prostaglandin D2-induced sleep, and its receptor has an important role in driving sleep [42]. In addition, the active components contain various *Ganoderma* triterpenes; some act on the same target genes (e.g., TNF and NR3C2). This synergistic effect may strengthen interventions against the target gene.

A PPI network topology analysis yielded the core target genes in the *Ganoderma* protein interaction network, including TNF, CASP3, JUN, NR3C1, and HSP90αA1. Sleep and immunity are mutually regulated. TNF and HSP90αA1, two important factors in the immune system, are closely related to sleep regulation [43]. The proinflammatory cytokine TNF increases non-rapid eye movement sleep [29]. HSP90αA1 participates in cell cycle regulation and signal transduction; it also mediates inflammatory responses and apoptosis [44]. CASP3, JUN, and other core proteins are closely related to apoptosis, which may involve TNF [45], although the underlying mechanism is unclear. In summary, the effect of *Ganoderma* on insomnia is at least partly mediated by immunity and apoptosis, although this hypothesis should be confirmed by KEGG analysis.

The GO results showed that *Ganoderma* modulates mainly circulatory metabolism, synaptic signalling, cell secretion and response, G protein-coupled receptor signalling, and other categories; these results indicated effects on various biological processes, among which biological rhythm is most closely related to insomnia. The KEGG enrichment results showed that during the treatment of insomnia by *Ganoderma*, the neuroactive ligand-receptor interaction signalling pathway is active; furthermore, core target genes (e.g., TNF, JUN, and HSP90αA1) regulate the calcium, apoptosis, cAMP, PI3K/Akt, and TNF signalling pathways, thereby modulating apoptosis/survival and the expression of various cytokines. Calcium signal transduction in astrocytes is reduced during sleep; it is involved in the regulation of slow-wave sleep [46]. Moreover, calcium signalling is important in the apoptosis pathway, and there are interactions between these pathways [47]. Therefore, calcium signalling may have a key role in the regulation of insomnia by *Ganoderma*. Cholinergic and serotonergic synapses are also related to insomnia [30, 31] and have important regulatory roles in both sleep and arousal; they may be targets of *Ganoderma*.

There was a high degree of cross-correlation among central active components, other components, and target genes of *Ganoderma*. In the central mechanism, *Ganoderma* mainly affects target genes in the pineal body, amygdala nucleus, prefrontal cortex, cerebellum, and other regions, which regulate rhythm-related physiological processes. In the peripheral mechanism, *Ganoderma* mainly affects target genes in the heart, thyroid gland, liver, and other organs. The target genes of active components were highly expressed in the central and peripheral organs, consistent with the important roles of active components. Therefore, *Ganoderma* ameliorated insomnia by regulating central and peripheral mechanisms.

The reverse disease enrichment results showed that *Ganoderma* has potential as an adjuvant treatment for insomnia or for treating neurological, cardiovascular, cerebrovascular, and digestive system diseases through the extensive pharmacological activities of the triterpenoid and sterol components. There is a bidirectional relationship between the above diseases and insomnia. Accordingly, the above diseases may change organism status and affect sleep. Therefore, *Ganoderma* can carry out bidirectional intervention on insomnia and its complications in the treatment of insomnia. This therapeutic advantage is a result of multi-component and -target TCM. There may be active components and targets in TCMs that have not been experimentally verified, suggesting that additional pharmacological mechanisms should be identified. The mechanism of action of *Ganoderma* must be verified in various models, including—but not limited to—animal, network, and multi-view models*.* In the future, updating and optimisation of artificial intelligence algorithms and their fusion with multi-modal data can provide new approaches to assess the molecular mechanisms of action of TCMs with multiple components and targets.

## Conclusions

*Ganoderma* is rich in multiple active components, corresponding to a considerable number of target genes. On this basis, *Ganoderma* intervenes in various biological processes and signalling pathways. Macroscopically, *Ganoderma* intervenes in the central mechanism (pineal body, amygdala nucleus, prefrontal cortex, and cerebellum) and peripheral mechanism (heart, thyroid gland, and liver) to ameliorate insomnia. In terms of the pharmacological mechanism, *Ganoderma* induces immune regulation, cell apoptosis/survival, and cell cycle regulation; it may affect biological processes such as circulatory metabolism, synaptic signal regulation, and rhythm regulation. There is a high degree of cross-correlation among the components, target genes, and target organs of *Ganoderma*, which provides a scientific explanation for its pharmacological activities. The findings of this study provide a reference for determining the mechanism underlying the effect of *Ganoderma* on insomnia and offer insights for future research.

## Data Availability

The datasets used and/or analyzed during the current study available from the corresponding author on reasonable request.

## Abbreviations

PPI: Protein-protein interaction
GO: Gene Ontology
KEGG: Kyoto Encyclopedia of Genes and Genomes
TCM: Traditional Chinese medicine
TNF: Tumor necrosis factor
2D: Two-dimensional
3D: Three-dimensional
TCMIP: Integrative Pharmacology-based Research Platform of Traditional Chinese Medicine
QED: Quantitative Estimation of Drug-likeness
TCMSP: Traditional Chinese Medicine Systems Pharmacology Database and Analysis Platform
OB: Oral bioavailability
DL: Drug-likeness
BBB: Blood-brain barrier
ASPL: Average Shortest Path Length
BC: Betweenness centrality
CC: Closeness centrality
CASP3: Caspase-3
JUN: Transcription factor AP-1
NR3C1: Glucocorticoid receptor
HSP90αA1: Heat shock protein HSP 90-alpha
NR3C2: Mineralocorticoid recepto
PI3K-Akt: Phosphatidylinositol 3-kinase-protein kinase B
IL-17: Interleukin 17
TNF-α: Tumor necrosis factor-alpha
TNF-R: Tumor necrosis factor-Receptor
ER: Estrogen receptor
GPCR: G protein-coupled receptor
CASP: Caspase
PRKCA: Protein kinase C alpha type
BAX: Apoptosis regulator BAX
COX2: Cytochrome c oxidase subunit 2
TGFB1: Transforming growth factor beta-1
cAMP: Cyclic adenosine monophosphate
PKA: Protein kinase A
PDE3A: Phosphodiesterase 3A
PI3K: Phosphatidylinositol 3-kinase
AKT(PKB): Kinase-protein kinase B
BCL2: B-cell lymphoma 2
NOS2: Nitric oxide synthase
